# Surgical identification of brain tumour margins through impedance monitoring and electrocorticography and the potential for their combined use: A systematic review

**DOI:** 10.1007/s10143-024-03134-0

**Published:** 2024-12-06

**Authors:** Ariadni Georgiannakis, Christopher A. R. Chapman, Dimitrios Paraskevopoulos

**Affiliations:** 1 Blizard Institute, Queen Mary University of London, London, UK; 2 School of Engineering and Materials Science, Queen Mary University of London, London, UK; 3 Department of Neurosurgery, The Royal London Hospital, Barts Health NHS Trust, London, UK

**Keywords:** Glioma, Brain tumour, Electrocorticography, Impedance, Resistivity

## Abstract

**Context:**

Primary central nervous system tumours have poor survival outcomes. Surgery, the first-line treatment, presents technical limitations, such as visualising the whole tumour border. Intracranial impedance monitoring and electrocorticography techniques provide insights into the local field potential characteristics, resistance and capacitance properties of brain tissue. We hypothesised that measurements obtained by either modality can distinguish between tumour and healthy brain tissue intraoperatively.

**Methods:**

A “Preferred Reporting Items for Systematic Reviews and Meta-Analyses” (PRISMA)-compliant systematic review was conducted, searching PubMed, Ovid, Scopus, Cochrane and Web of Science. Studies on electrocorticography and impedance monitoring in patients with brain tumours were included. Data on patient demographics, technical details, obtained results and safety were extracted and analysed in Excel.

**Results:**

Eighteen studies involving 286 patients in total were identified. Ten impedance studies showed that brain tumour tissue has significantly different values than healthy tissue, while its resistivity varies, being either higher or lower. Eight electrocorticography studies indicated increased high gamma power and altered connectivity in tumour tissue. No studies integrated impedance monitoring and electrocorticography in one device.

**Conclusion:**

Impedance and electrocorticography measurements have the potential of differentiating between tumour and unaffected issues intra-operatively. Larger studies with standardised protocols are needed to validate these findings. Additionally, the combination of these two modalities has the potential for improved specificity with a single device. Future research should explore the role of these modalities in enhancing tumour margin identification across different tumour subtypes and in improving survival outcomes.

**Supplementary Information:**

The online version contains supplementary material available at 10.1007/s10143-024-03134-0.

## Introduction

Primary brain and other central nervous system (CNS) tumours affect more than 12,500 patients annually in the United Kingdom (UK) and survival rates are low, with 31% of patients with glioblastoma (GBM) reaching a 2-year survival and only 17% surviving past 5 years [1]. The 2021 World Health Organisation (WHO) classification of CNS tumours recognises over 100 tumour types [2], the heterogeneity of which has hampered the success of previous treatments [3].

Currently, the European Association of Neuro-Oncology (EANO) guidelines, recommend the intent of surgery for patients with glioma [4]. The effectiveness of surgical resection for brain tumours is often constrained by their location, for example when they are situated near vital or eloquent structures [5] or in deeper areas [6]. Another issue is the diffuse glioma borders making it challenging to distinguish and remove the entire tumour [4, 7, 8]. Furthermore, following craniotomy and dural opening, “brain-shift” may reduce the effectiveness of pre-operative images for surgical guidance [9, 10].

### Tools used for surgical guidance

To overcome surgical challenges, several tools and imaging methods are used routinely to improve surgical outcomes. These include 5-Aminolevulinic Acid (5-ALA) fluorescence guidance [11], neuro-navigation [12], intra-operative Magnetic Resonance Imaging (iMRI) [13] and intra-operative Ultrasound (iUS) [14]. Despite their widespread adoption [15, 16], they largely rely on subjective visual-feedback [11, 12], can be costly [13] and are operator dependent [14, 17].

Moreover, many novel modalities such as Raman spectroscopy [18] are being actively developed to circumvent some of the limitations of current tools [18, 19]. However, the majority of these are still at the experimental pre-clinical phase and are focused on tissue biopsies. Additionally, they often only provide measurements at one point in time (i.e. intra-operatively) and thus do not allow for continuous monitoring of the tumour resection cavity [20]. Therefore, despite the high spatial resolution of current techniques, identifying signals of a higher temporal resolution, allowing intra- and post-operative monitoring could enhance the extent of resection, which proportionately relates to survival [21], and supplement the monitoring of CNS tumours following surgical intervention [20].

### Bioimpedance monitoring

**Fig. 1:**
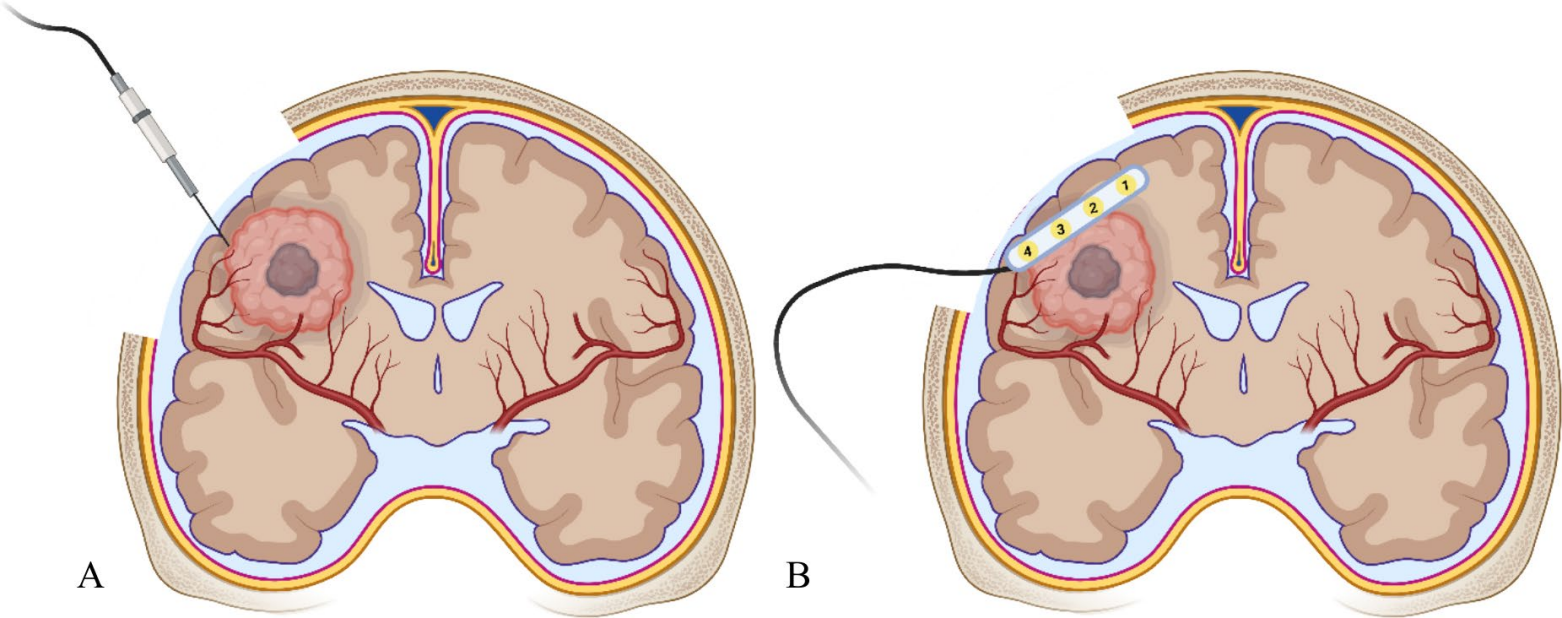
Simplified visualisation of impedance measurement with a monopolar probe (**A**) and electrocorticography with 1 × 4 electrode strip (**B**) on an intra-axial brain tumour of the right frontal lobe (own work). Created with BioRender.com

Bioimpedance describes the electrical properties of biological tissue as current flows through it [22]. In the neurosurgical setting, impedance has been previously measured in patients with epilepsy, using multi-contact electrodes, with epileptogenic tissue presenting a lower impedance than healthy brain matter [23] (Fig. 1A).

### Electrocorticography

Electrocorticography (ECoG) is a type of invasive electroencephalography (EEG), whereby electrodes are placed directly on the exposed surface of the brain to record electrical activity form the cerebral cortex [24] (Fig. 1B). Currently, it is used for intraoperative neurophysiology monitoring in epilepsy surgery [25] and to identify intraoperative seizures and after-discharges associated with cortical stimulation of functional areas during tumour resection [26].

Leveraging the electrical properties of brain tumour tissue has the potential to rapidly and continuously identify tumour regions. The restructuring of the neural tissue in a glioma have been shown to express differences in tissue bioimpedance [27] and changes in tissue excitability [28, 29]. Additionally, impedance monitoring and ECoG use similar hardware and are currently employed safely in neurosurgical procedures so they present an attractive cost-effective solution for continuous differentiation between healthy and tumour tissue.

This systematic review studied the use of ECoG and impedance measurement in brain tumour surgery existing in the literature. We assessed whether the local field potential characteristics and the impedance (or resistivity) of tumoural, peri-tumoural and apparent healthy brain tissue differ; and explored if these differences could be measured intraoperatively by ECoG and impedance electrodes, either separately or through a single device.

## Methods

The protocol for this systematic review was registered on PROSPERO (CRD42024528246) and the Preferred Reporting Items for Systematic Reviews and Meta-Analyses (PRISMA) guidelines [30] were followed.

The review question was defined using the Population, Intervention, Comparator, Outcome (PICO) framework. The population was patients with primary or secondary brain tumours, the intervention was either ECoG or intracranial impedance monitoring and the outcome was frequency (Hz) and resistance (Ohms or Ω) measurements as well as the safety and effectiveness of the interventions. The search strategy was developed with the QMUL librarians. We searched the databases PubMed, Ovid, Scopus, Cochrane and Web of Science for articles containing terms “glioma”, “brain tumour”, “electrocorticography” and “impedance” from inception to May 17, 2024. A filter to include publications in English only was applied with no other automation tools applied at any stage of the review process. The full search strategy is available in Appendix 1. Since the interventions reviewed are not routinely used in brain tumour surgery, we also performed additional citation searches of included reports and conducted a search on Google Scholar with the key terms to identify any omitted publications. Searches were re-run after the review was completed and a supplementary search on ClinicalTrials.gov was conducted to ongoing studies that are of interest to the review question.

Identified publications were added to Rayyan [31] and after deduplication, the titles and abstracts of all articles were screened for eligibility by AG and CC in a blinded fashion. Any conflicts were resolved following discussion between the authors. The final list of included articles was reviewed by senior author DP. The detailed inclusion and exclusion criteria, in accordance with the PICO question, that were applied during the screening process are found in Table 1. Of note, interventions for tumour-related epilepsy were excluded if they did not describe differences between tumour and healthy tissue as one of their outcomes.

**Table 1:** Inclusion and exclusion criteria for studies

Inclusion criteria	Exclusion criteria
• Original research articles (i.e.randomised control trials, cohort, case–control and cross-sectional studies) • Adults and children • Any type of primary or secondary brain tumour • Patients undergoing ECoG and/or impedance monitoring intervention during surgical treatment	**•** Review articles, editorials, conference proceedings **•** Animal, ex-vivo and in-vitro studies **•** Interventions for tumour-related epilepsy **•** Non-English language **•** Patients undergoing surgical or conservative treatment alone

A risk of bias assessment was undertaken independently by AG and CC using the ROBINS-I tool [32] which is supported by the latest Cochrane recommendations [33]. Disagreements were reviewed by senior author DP. Results were represented using the robvis tool [34].

Extracted data included: patient demographics, technical aspects of ECoG and impedance monitoring, obtained measurements for each tissue type (tumour, peri-tumour and healthy tissue) and any procedure-related complications. Where the data was missing, or information was unclear this has been accounted for (described as “not reported”). Data was collected by AG and was independently reviewed by DP and CC.

The data was analysed and presented using Microsoft Excel. Descriptive statistics were used for patient characteristics. As there was considerable variation in study designs and reporting of outcomes, data was mainly synthesised qualitatively. For impedance studies, a meta-analysis of available Individual Patient Data (IPD) was conducted in order to assess measurements across a larger number of patients and tissue types. The means and standard deviations for the impedance of each tissue type for every patient were calculated, if not directly provided. IPD distribution was tested for normality using the Shapiro–Wilk test and a two-tailed Mann–Whitney U Test (Wilcoxon Rank Sum Test) was performed in IBM SPSS Statistics (version 29) to compare the measured impedance values across tissue types. Where IPD was not available, we extracted the in-text reported means and standard deviations.

## Results

### Search results

**Fig. 2:**
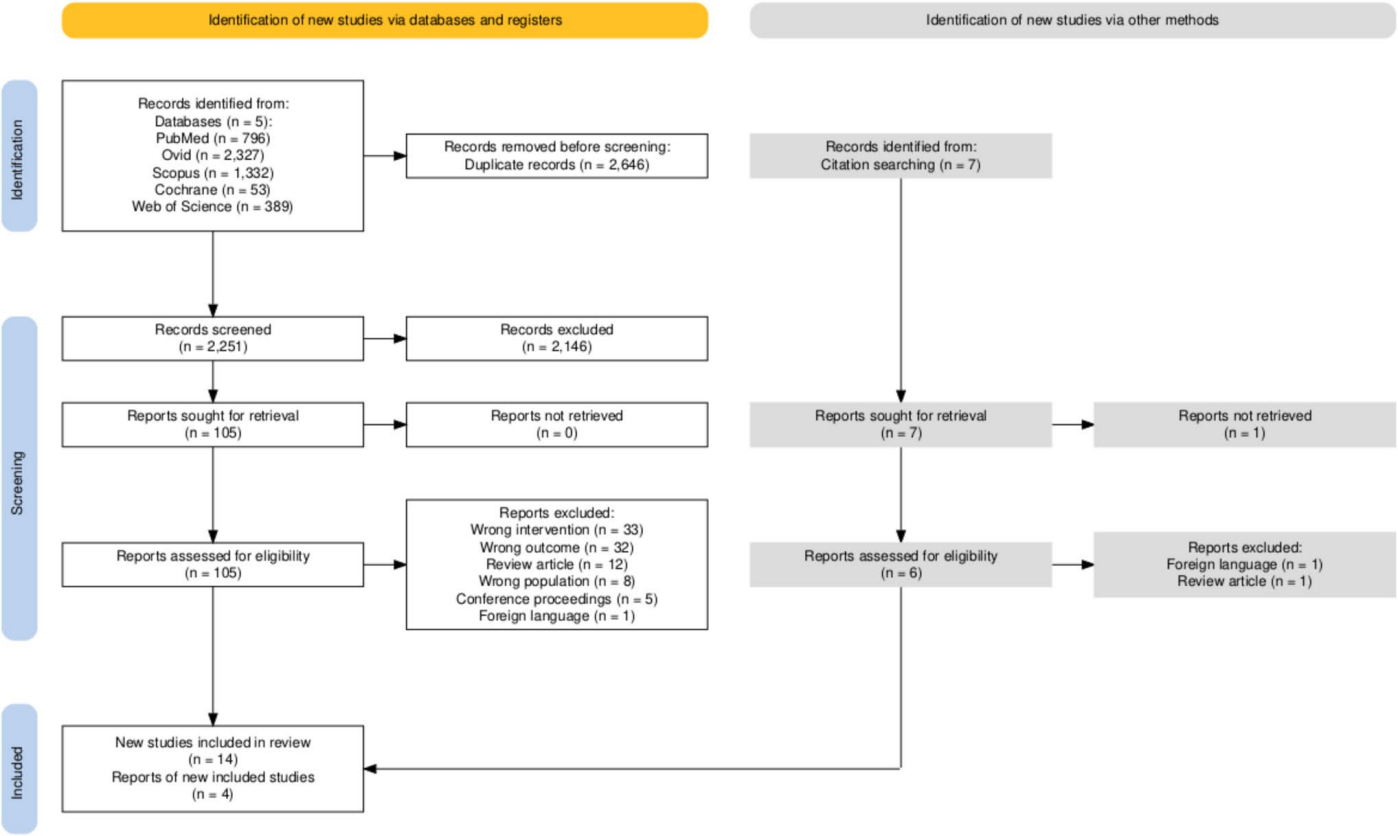
PRISMA systematic review flowchart of screening

Our search yielded 2,251 unique articles regarding the use impedance probes or ECoG in intraoperative brain tumour monitoring. Seven further articles were found via citation searching of included articles. Following application of the exclusion criteria, a total of 18 articles were included in our study, of which 10 were specific for impedance [35–44] and 8 for ECoG [29, 45–51] (Fig. 2). This provided details of 286 different patients, 216 of whom had impedance monitoring and 70 of whom had intraoperative ECoG. No study exploring measurement of impedance and electrical signature using a single device (i.e. ECoG) was identified. Regarding design, all research was conducted as small-scale prospective experimental feasibility studies.

### Risk of bias assessment

**Fig. 3:**
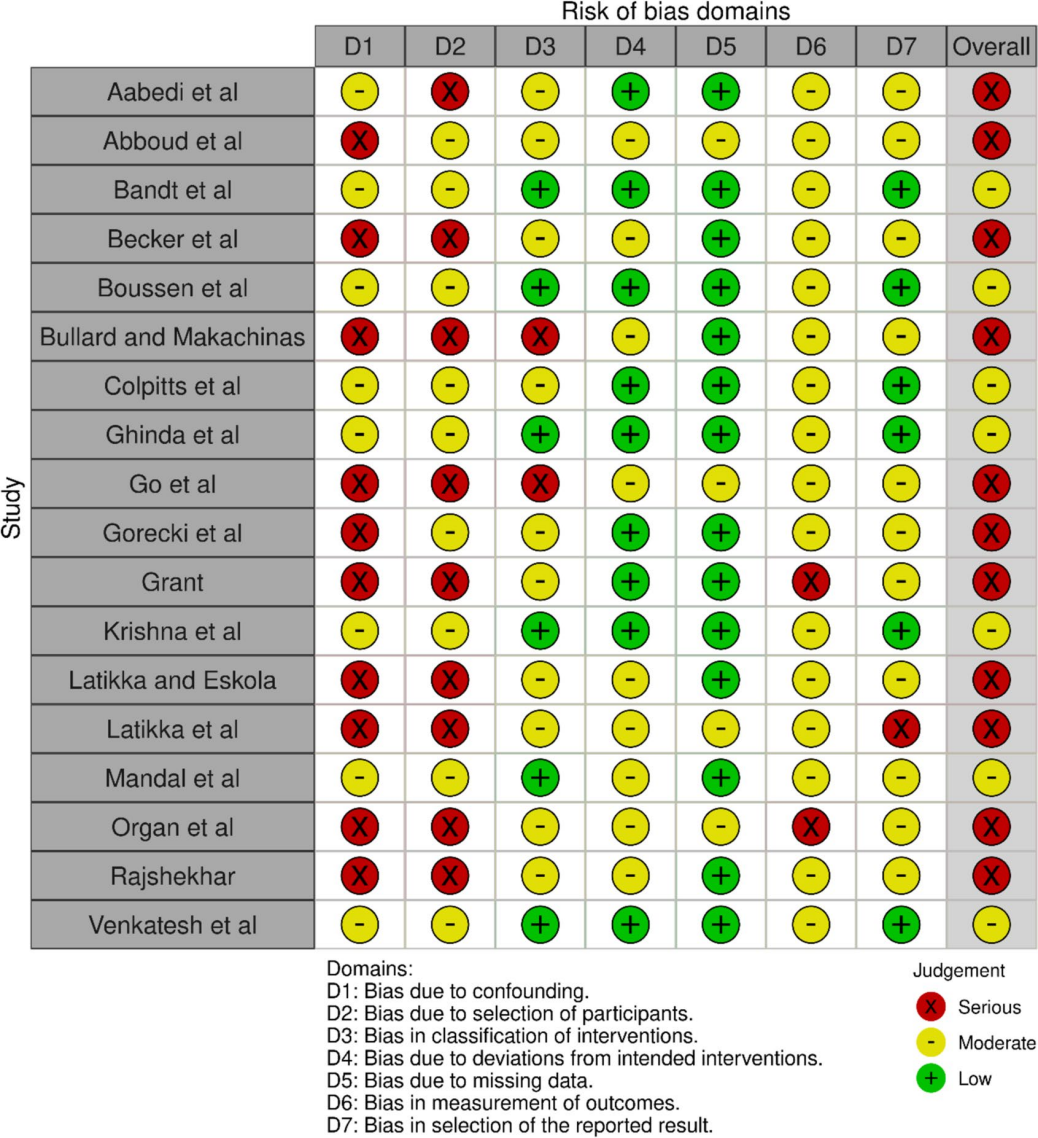
Detailed Risk of Bias (RoB) analysis results using the ROBINS-I tool. This graph was created using the robvis tool

Risk of bias analysis results are available in full in Fig. 3, with 11 studies scoring as “high risk”, 7 studies presenting “some concerns” and no studies being of “critical” or “low risk”. Although all study designs were prospective and some had pre-registered protocols available, this is a relatively small field of research without established best practices.

### Impedance results

The patient and tumour characteristics can be seen in Table 2. In general, study aims were to investigate the feasibility of in-vivo measurement of impedance during brain surgery and to assess whether there is a difference between the electrical impedance of brain tumours and apparent healthy tissue. Where reported, ages ranged from 18 to 88 years and there were more males (*n* = 63, 57.8%) than females. Additionally, there was a similar distribution of tumours located in either the left or right hemisphere (9 in the left and 6 in the right). Reported pathologies studied included intra- (*n* = 150, 70.0%) and extra-axial (*n* = 31, 14.5%) brain tumours and CNS metastases (*n* = 33, 15.5%). Most of the WHO grades of tumours corresponded to high grade gliomas (HGG) (*n* = 64, 67.3% grades III-IV). No further information on the molecular profile of lesions was available.

**Table 2:** Impedance monitoring study and patient characteristics

Author	Year	Country	Patient (n)	Mean Age (range)	Male to female ratio	Pathology	Tumour laterality (n)	WHO grade (n)	Tumour location (n)
Abboud et al. [35]	2021	Germany	92	60.0 (18–88)	52:40	GBM (33), Metastasis (33), LGG (16), HGG (10)	NR	II (16), III (10), IV (33), NR (33)	Frontal (41), Parietal (20), Temporal (15), Occipital (8), Insula (8)
Becker et al. [36]	1970	United States	4	NR	NR	HGG (2), GBM (1), Meningioma (1)	NR	III (2), IV (1), NR (1)	NR
Bullard and Mackachinas [37]	1987	United States	19	NR	NR	GBM (11), HGG (4), LGG (3), Lymphoma (1)	NR	NR	Frontal (5), Basal ganglia (4), Corpus callosum (1), Parietal (7), Thalamus (2)
Go et al. [38]	1972	The Netherlands	9	45.4 (22–70)	5:4	HGG (6), LGG (2), GBM (1)	Left (5), Right (4)	II (2), III (6), IV (1)	Temporal (2), Frontal (5), Parietal (1), Occipital (1)
Gorecki et al. [39]	1990	Canada	13	NR	NR	Glioma (10), Craniopharyngioma (2), Lymphoma (1)	NR	NR	NR
Grant [40]	1926	United States	12	NR	NR	Endothelioma (5)*, Glioma (5), Sarcoma (1), Meningioma (1)	NR	NR	Frontal (6), Motor cortex (2), Temporal (2), Occipital (1), Parietal (1)
Latikka et al. [41]	2001	Finland	8	59.7, (32–87)	6:2	Meningioma (3), GBM (2), Cystic tumour (1), tumour (1)**, glioma (1)**	Left (2), Right (1), NR (5)	NR	NR
Latikka and Eskola [42]	2019	Finland	18	55.8 (4–77)	NR	HGG (5), LGG (4), Meningioma (8), Pinealoma (1)	NR	NR	NR
Organ et al. [43]	1968	Canada	14	NR	NR	Metastasis (2), Meningioma (5), GBM (3), HGG (4)	Left (2), Right (1), NR (11)	NR	NR
Rajshekhar [44]	1992	India	27	NR	NR	LGG (13), HGG (9), Craniopharyngioma (3), Lymphoma (1), Pinealoma (1)	NR	I (4), II (9), III (6), IV (1), NR (7)	NR

*NR*: Not Reported, *HGG*: High Grade Glioma (WHO grade ≥ III), *LGG*: Low Grade Glioma

*As this was an earlier study, it is assumed that “endothelioma” refers to what would now be classified as meningioma [52]

**Exact type of brain tumour and glioma not specified in text

Six studies opted for a transcranial approach [35, 36, 38, 40–42], while the remaining four conducted a stereotactic operation [37, 39, 43, 44]. Of note, four studies reported continuous monitoring as the probe was transversing different tissues [38, 39, 43, 44] while the rest reported single point measurements either before or after the tumour resection [35–37, 40–42]. No impedance studies reported on their anaesthetic regimens, or whether these were changed compared to standard practice.

The choice between type of probe varied, with six studies using a monopolar, three studies using a bipolar and one study using a custom-made 3 electrode probe (Table 3). Where reported, the location of the probe was confirmed intra-operatively in three studies either via imaging (CT/MRI) or 5-ALA enhancement [35, 39, 44]. Furthermore, three studies compared impedance measurements with imaging or biopsy findings [37–39]. Where reported, the electrode tip lengths were 2–5 mm [36–39, 41, 42, 44], with their diameters ranging from 1–3 mm [35, 37–39, 41, 42, 44] and only one study reported the inter-tip distance, which was 5 mm [35]. The materials of the electrodes used were mostly steel [35–38, 43] or platinum iridium [40].

**Table 3:** Impedance monitoring results

Study	Probe characteristics	Total measurements per region (n)	Tumour recordings (n)	WM mean resistivity/impedance	GM mean resistivity/impedance	Peri-tumoural mean resistivity/impedance	Tumoural mean resistivity/impedance	Impedance/resistivity ratio	Additional findings
Abboud et al. (2021) [35]	Bipolar	WM: 32, oedema: 14, necrotic area: 63	58	13.3 ± 1.7Ωm (9–18)*	NR	NR	CT enhancing areas: 5.1 ± 1.7Ωm (2.8–7.8), Non-CT enhancing areas: 6.3 ± 1.1Ωm (4.5–8.4)*	NR	Oedema: 8.5 ± 1.6Ωm (6.7–10.1), necrotic areas: 4 ± 1.2Ωm (1.6–8.3)*
Becker et al. (1970) [36]	Monopolar	NR	NR	NR	NR	NR	318.75 ± 85.48Ω (240–425Ω)	0.44 ± 0.19 (0.25–0.71)	NR
Bullard and Mackachinas (1987) [37]	Monopolar	WM: 24, GM: 27, peritumoural area: 22	27	430.22 ± 220.60Ω (150–800)	395.19 ± 169.02Ω (150–700)	408.10 ± 218.34Ω (100–850)	382.50 ± 190.58Ω (100–800)	NR	NR
Go et al. (1972) [38]	Monopolar	WM: 3, GM: 5, haemorrhagic areas: 7	8	220 ± 62.45Ω (150–270)	380.00 ± 76.16Ω (270–470)	NR	WM: 290.83 ± 88.68Ω (220–400), GM: 280 ± 155.56Ω (170–390)	NR	NR
Gorecki et al. (1990) [39]	Bipolar	NR	NR	500–600Ω*	600–700Ω*	NR	NR	NR	CSF: 300–400Ω, Thalamus/basal ganglia: 450–550Ω*
Grant (1926) [40]	Bipolar	NR	NR	NR	602.08 ± 128.12Ω (525–900)	NR	429.54 ± 399.34Ω (200–1600)	NR	NR
Latikka et al. (2001) [41]	Monopolar	WM: 21, GM: 29, CSF: 2	NR	3.91Ωm (3.26–4.77)	3.32Ωm (1.94–5.41)	NR	2.3–9.7Ωm	NR	CSF: 0.8Ωm
Latikka and Eskola (2019) [42]	Monopolar	WM: 28, GM: 36	92	3.72 ± 0.93Ωm (2.49–4.98)	2.91 ± 1.26Ωm (1.30–5.30)	NR	4.23 ± 1.99 Ωm (0.73–8.56)	3.72 (0.43–3.30)	NR
Organ et al. (1968) [43]	Custom three electrode probe	NR	NR	1440 ± 155Ω	925.52 ± 396.56Ω	NR	880.77 ± 616.62Ω (350–1900)	0.75 (0.32–1.6)	CSF: 650 ± 230Ω
Rajshekhar (1992) [44]	Monopolar	WM: 39, GM: 39, peri-tumoural area: 34	39	686.54 ± 318.60Ω (300–2000)	674.36 ± 178.49Ω (325–1500)	513.97 ± 113.68Ω (350–850)	521.79 ± 154.66Ω (300–950)	NR	NR

*GM:* Grey Matter, *WM*: White Matter, *NR*: Not Reported, *Ωm*: Ohm meters,* Ω*: Ohms, *CT*: Computerised Tomography, *HGG*: High Grade Glioma (WHO grade ≥ III), *LGG*: Low Grade Glioma

*Values directly extracted from published study manuscript as IPD not available

The current signal and frequencies applied varied across studies. For example, Organ et al. applied a current of 0.1 μΑ at a frequency of 100 Hz [43], Abboud et al. injected 0.7 μΑ at 140 Hz [35], Go et al. used 1 μΑ at 10 kHz [38], both studies by Latikka et al. operated with 2 μΑ at 50 kHz [41, 42] and Becker et al. injected currents at 20 mA [36]. The low currents used are in line with the use of small surface area of the probes. Lastly, five studies reported calibration of the probe before measurements were obtained [35, 36, 40–42].

Key results from impedance monitoring studies are highlighted in Table 3. The average total number of recordings of the healthy WM was 25, for healthy cortical tissue this was 27 and 45 for pathologic tissues. Only one study measured the impedance of associated oedema and necrotic areas [35] and three measured the impedance of peri-tumoural areas, obtaining an average of 21 measurements. Where reported, characterisation of tissue was based on intra-operative MRI-based neuronavigation with or without 5-ALA enhancement [35, 39], CT [35, 37, 39, 44] or intraoperative histology [37, 38].

**Fig. 4:**
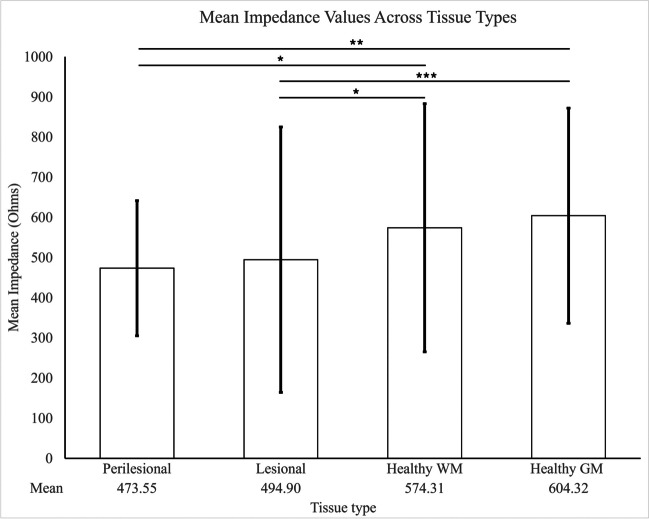
Pooled means of impedance measurements from different tissue types. Tissue from the perilesional area had the lowest impedance followed by lesional tissue, healthy white matter (WM) and lastly, healthy grey matter (GM) [36–38, 40, 43, 44]. There was a statistically significant difference between perilesional and healthy WM tissue and between lesional and healthy WM tissue at *p < 0.05. The difference between perilesional and healthy GM tissue was significant at **p < 0.01, and the difference between lesional and healthy GM tissue was significant at ***p < 0.0001 (Mann Whitney U Test). There was no statistically significant difference between perilesional and lesional tissue. (Own work)

Impedance measurements were reported either as Ohms (Ω) which is a direct measure of impedance in seven studies [36–40, 43, 44] or Ohm meters (Ωm), which refers to the resistivity of tissue (does not account for resistance and reactance), in three studies [35, 41, 42]. Individual means reported by each study can be seen in Table 3. A pooled mean impedance of tissues was calculated based on the IPD of six studies [36–38, 40, 43, 44]. This was calculated to be 574.31 ± 308.62 Ω in white matter (WM) and that of grey matter (GM) was 604.32 ± 267.94 Ω. The mean impedance of perilesional areas was noted to be 473.55 ± 168.03 Ω and that of lesions was 494.90 ± 330.45 Ω. Statistically significant differences were observed between all tissue types except for between perilesional and lesional tissue (Fig. 4).

In general, it was observed that resistivity and impedance values fell when the probe entered the lesion [53] and high-grade gliomas had higher impedance values than lower grade gliomas [35].

No studies reported on surgical outcomes such as extent of resection of the lesion, however, two separate studies reported on the safety of invasive impedance monitoring. Complications included intraoperative haemorrhage due to the probe passing through a sulcus in one patient [37], transient worsening of pre-existing hemiparesis in three patients, and a small haematoma in one patient [39]. However, these issues resolved, and no deaths were reported. Issues were observed in earlier studies, whereas later studies did not report any risks, potentially because probes used were the same as those routinely used for treating pain disorders [41, 42].

No studies reported on surgical outcomes such as extent of resection of the lesion, however, two separate studies reported on the safety of invasive impedance monitoring. Complications included intraoperative haemorrhage due to the probe passing through a sulcus in one patient [37], transient worsening of pre-existing hemiparesis in three patients, and a small haematoma in one patient [39]. However, these issues resolved, and no deaths were reported. Issues were observed in earlier studies, whereas later studies did not report any risks, potentially because probes used were the same as those routinely used for treating pain disorders [41, 42].

### Electrocorticography results

The main patient characteristics and pathologies of ECoG studies are shown in Table 4. The studies aimed to define how neural circuits are altered in the presence of brain tumours [45–47, 49–51], with some observing the hyperexcitable environment CNS tumours induce [29] and one exploring its relationship with spreading depolarisation (SD) [48]. The mean age was 48.9 (range: 19–78) and the proportion of males and females was similar (males:females, 29:26). Furthermore, most tumours were found in the left hemisphere (*n* = 41, 70.7%), while the specific lobes affected differed. Tumour pathologies included various gliomas and only one metastasis [47]. WHO grades varied, although most patients had a high-grade tumour (Grade II: 21, II-III: 8, III: 15, IV: 22). Compared with the impedance studies, molecular phenotypes were more extensively described, as five studies included the IDH-1 mutation, MGMT methylation, 1p/19q and/or ATRX mutation statuses [29, 45, 48, 49, 51]. The mean maximal tumour diameter was only provided in one study and was 6.63 mm (range, 4–11 mm) [48]. Only one study reported on the presence of previous cranial procedures [47] and two studies reported concurrent pharmacological treatment of co-morbid epilepsy [48, 49].

**Table 4:** Electrocorticography study and patient characteristics

Author	Year	Country	Patient (n)	Mean age (range)	Male to female ratio	Pathology type (n)	Tumour laterality (n)	WHO grade (n)	Tumour location (n)
Aabedi et al. [45]	2021	United States	12	49.9 (19–78)	10:2	HGG (6), LGG (3), GBM (3)	Left (12)	II (3), III (5), IV (4)	Perisylvian (12)
Bandt et al. [46]	2017	United States	6	61.2 (48–77)	2:4	GBM (6)	Left (6)	IV (6)	Temporal (3), Parietal (2), Temporo-occipital (1)
Boussen et al. [47]	2016	France	16	42.7 (20–74)	7:9	LGG (8), HGG (5), Metastasis (1), Ganglioglioma (1), GBM (1)	Left (2), Right (14)	II (8), III (4), IV (1)	Parietal (2), Temporal (2), Frontal (3), Frontotemporoparietal (1), Opercula (1), Frontoinsular (1), Cingulum (1), Insula (2), Temporoinsular (2), Temporoparietal (1)
Colpitts et al. [48]	2022	United States	3	NR	NR	GBM (3)	Left (1), Right (2)	IV (3)	Temporal (1), Frontal (1), Parietal (1)
Ghinda et al. [49]	2020	Canada	12	41 (29–52)	5:7	LGG (8), HGG (4)	NR	II (8), III (2), IV (2)	Frontal (7), Parietal (1), Frontal-insular (1), Temporal-insular (1), Frontotemporal (1), Frontoparietal (1)
Krishna et al. [50]	2023	United States	14	NR	NR	Oligodendroglioma (4), Astrocytoma (4), GBM (4), “Other glioma” (2)	Left (14)	II-III (8), IV (4), other (2)	Frontal (14)
Mandal et al. [51]	2024	United Kingdom	4	35 (29–41)	2:2	LGG (2), HGG (2)	Left (3), Right (1)	II (2), III (2)	Frontal (3), Temporal (1)
Venkatesh et al. [29]	2019	United States	3	64.66 (57–74)	3:0	HGG (1), GBM (2)	Left (3)	III (1), IV (2)	Parietal (1), Temporal (2)

*NR*: Not Reported, *GBM*: Glioblastoma, *HGG*: High Grade Glioma (WHO grade ≥ III), *LGG*: Low Grade Glioma

Most measurements were obtained during awake surgery with some stimulus (usually verbal), however two studies made recordings in the asleep phase [46, 48], and one took measurements during both asleep and awake stages [49]. The anaesthetic regiments used in electrocorticography studies included remifentanil and propofol, the latter of which was weaned off for around 20 min before the awake measurements were obtained [29, 45–47, 50, 51].

All patients had transcranial surgery, and, in most studies (*n* = 7, 87.5%), subdural electrodes were applied on the pre-resection window with their placement confirmed through visual analysis, intra-operative photography and neuronavigation [29, 45–47, 49–51]. The mean duration of intraoperative ECoG recordings was 1.76 min (range: 0.067–5 min) [29, 45–47, 49–51]. In one study, the subdural electrodes were applied following tumour resection and were left inside the tumour cavity post-operatively for longer-term observation (mean: 36.34 h, range: 23- 59.8 h). The position of these electrodes was confirmed via post-operative CT imaging [48].

The sizes of ECoG grids varied, with one study using as little as 4 electrodes and another up to 96 electrodes [45, 51]. Additionally, some studies reported to use custom grids depending on the tumour size [49, 50]. Only three studies reported the mean number of ECoG channels per tissue type, which ranged from 8–9 for healthy tissue, 9–17 for peri-tumoural tissue and 10–16 for tumour tissue [29, 49, 50]. Where reported, the electrode diameter ranged from 0.5–5 mm [46, 47, 49, 51] and the interelectrode distance was 10 mm [45–47, 51]. The materials of electrodes, where reported were platinum [45–47].

Generally, tumour tissue was defined as that presenting a macroscopic visual suspicious appearance and was confirmed with signal changes on T1-contrast or FLAIR MRI [29, 45, 47]. The peri-tumoural zone was defined by studies to start at least 10 mm away from the tumour boundaries, also based on imaging [46, 50], while Venkatesh et al. determined the infiltrative margin of the tumour to correspond to the enhancing rim of the tumour as seen on T1-contrast MRI [29]. More specifically, Ghinda et al. used the Response Assessment in Neuro-oncology (RANO) criteria to classify tissues [49]. Healthy tissue was determined to be that which was distal to the tumour tissue and/or did not demonstrate any abnormalities on MRI scans [29, 46, 50].

The variability of study design and reporting of outcomes meant that it was not possible to tabulate data, rather some key themes were observed:

- Tumour tissue exhibits a co-ordinated temporal increase in frequencies relating to a stimulus [45, 46, 50], and connectivity within the tumour is maintained [46] or increased in the cortical parts [47]. Bandt et al. described connectivity by calculating the temporal correlation of slow cortical potentials (< 0.5 Hz) between electrode pairs and used distance regression and topographical correlation to control for the effect of electrode proximity on connectivity [46]. Boussen et al. defined connectivity as the inter-relation between two distant electrodes and described it via linear and non-linear amplitude correlation, coherence (similarity in signal frequencies) and phase-locking (synchronisation in phase over time), assessing different aspects of connectivity but not accounting for spatial distance effects in measures [47].
- Spatially, diffuse planning sites are engaged beyond normally participating circuits [45, 50], which could indicate a degree of compensation.
- Tumour tissue also loses the ability to differentiate between more and less demanding cognitive tasks [45, 50].
- Tumour tissue represents consistent alterations in frequency powers, with some reporting a reduction in the overall power spectra of frequencies [46, 47] while others noting an increase in high gamma power [29, 50, 51].
- The encoding capacity of tumour cells (Shannon entropy) was noted to be reduced [45, 46].

In comparison, in distant apparent healthy cortex, no alterations were observed [45, 47, 49].

In the electrocorticography group, no complications at any point of the operation were reported. A potential explanation for this is that regular ECoG grids that are routinely used for mapping in oncology and epilepsy surgery were used in the studies, and therefore they have been previously tested for safety and sterility.

Our ClinicalTrial.gov search yielded two relevant registered studies at the recruitment phase (NCT05565118 and NCT06408428), in the United States and France. The first study will focus on correlating the pattern of electrical activity (hyperexcitability) of HGGs measured by ECoG, with their progression. The second study will employ micro-ECoG arrays, to investigate the relationship between recorded activity, tumour infiltration and oncometabolite concentration in diffuse gliomas.

## Discussion

Both impedance and ECoG studies showed that there was a difference in tumour and healthy tissue. The surgical approach and techniques for invasive impedance monitoring and performing ECoG varied largely across studies included in this systematic review, therefore, the generalisability of findings is significantly impacted by the experimental design and set outcomes.

The type of probe used in the studies often depended on availability and feasibility. Probes with fewer contacts are easier to manipulate intraoperatively, however, they are also more prone to influencing measured output due to improper calibration and electrode polarisation impedance, in turn impacting the accuracy and sensitivity of measurements [54, 55]. Moreover, frequencies and currents injected varied. Choosing an appropriate frequency for measuring the impedance of a neuronal membranes is important, as lower frequencies produce larger signals that are susceptible to artifacts, whereas higher frequencies provide smaller signals that are less affected [56].

To date, there remains no gold standard for impedance measurement of intracranial tissue and all described techniques in included articles are still at the experimental phase. Therefore, there are also no widely accepted “normative” values available for the electrical resistivity of brain and tumour tissue [57]. The studies included in this review demonstrated that healthy GM had the highest impedance values, followed by healthy WM, the tumoural zone and finally the peritumoural zone. Previous studies of breast cancer tissues have also shown that there are significant differences between tumour and healthy tissue although they note the variability and overlap of obtained values for each tissue type [58]. Additionally, a systematic review of 51 studies and 16 cancer subtypes studying impedance measurements of tumours in different organs, demonstrated that some tumours present higher impedance and some lower impedance than healthy tissue [59]. Similarly, a study on a porcine brain demonstrated that GM has a significantly lower impedance than WM which is in conflict with the findings of our pooled analysis but in agreement with the resistivity values reported in studies [60]. In another study of freshly excised GM and WM of patients with epilepsy undergoing surgery, the conductivity (which is the inverse of resistivity) of tissues with cortical dysplasia was higher than those without [61], which is in agreement with two of the resistivity studies included [35, 41].

It is unclear why the peritumoural zone has a lower impedance than the tumoural zone. Moreover, the definition of the peritumoural zone varied itself across studies. A potential reason for this difference could be due to the presence of vasogenic oedema, which as a fluid confers a lower impedance [35, 57].

The increasing number of recent studies in electrocorticography suggests growing interest and advancements in using ECoG for glioma research. Since ECoG has not been extensively studied for this indication, no conclusions can be made on best practices and expected measurements. The focus on the effect of gliomas on task-dependent neuronal activity in the high-gamma range is likely due to their location which is mainly near eloquent brain [62]. Interestingly, some studies reported an overall reduction in power spectra while others observed an increase in the high gamma power. Differences in the methodology used in each study could account for these differences; for example, Boussen et al. did not compute power spectra for high gamma activity whilst Venkatesh et al. and Krishna et al. only considered the high gamma range (> 70 Hz) [29, 47, 50]. Another possible reason for this could be due to measurements obtained at resting state, where gamma activity is supressed, or during task-related conditions, where it is increased. For example, Bandt et al. assessed activity at rest noting a decrease in gamma power while Venkatesh et al. and Krishna et al. demonstrated an increase in gamma activity during demanding tasks [29, 46, 50]. Furthermore, the variability in tumour grade and location could also impact the type of frequency power affected.

The included studies have shown that intracranial tumours behave as independent functional units that also engage parts of healthy brain [45–47, 50]. These findings could support the conduction of studies in pre-clinical glioma models to clarify the evolution of this phenotype and potential implications for treatment strategies [45]. Interestingly, the effect of SD that was noted by Colpitts et al. [48] has also been seen in an immunocompetent CRISPR GBM mouse model, where it was suggested that SD arising in the peri-tumoural region precedes tumour-related epileptogenesis, a common symptom in patients with GBM [63]. SD is also frequently observed in other intracranial pathologies such as stroke [64], so there may be interest in the long-term monitoring of SDs in the glioma resection bed to evaluate its role in disease propagation and recurrence [48].

Despite promising results, ECoG studies present some shortfalls. While the effect of anaesthesia on neuromonitoring is well recognised [65], this was only considered by Ghinda et al. who obtained measurements during both awake and asleep states [49]. Furthermore, the definition of the peri-tumoural area was not consistent across both ECoG and impedance studies, therefore conclusions specific to this transitional area cannot be drawn. Multimodal imaging including MRI, genomic and histological analysis could help circumvent this issue [66], however an objective reproducible characterisation of the peri-tumoural zone remains to be established [67].

The choice of ECoG strip or grid varied among studies and often was dependent on the tumour size. Similarly, impedance studies where an electrode probe was used meant that only specific points of different tissues were examined. This highlights an intrinsic limitation of the tools used in studies in this review regarding temporal resolution, compared to techniques such as 5-ALA that allow for synchronous visualisation of the tumour border. Potential directions for future studies would be to use electrode grids with multiple channels which can obtain more measurements at one-point in time along with higher quality observations due to more contact points [24]. Furthermore, custom-made grids could enable a more tailored approach and non-rectangular shaped grids, such as a circular grid may be more effective for tumour demarcation [68].

Given the categories of outcomes reported in both impedance and ECoG studies it was not possible to demonstrate the safety and effect of the intervention on survival, degree of resection, and morbidity. ECoG studies did not report on any complications and all the complications due to invasive impedance monitoring eventually resolved without causing mortality [37, 39]. As only two studies reported complications a full picture of the safety of this technique cannot be formed. We encourage future studies to assess and report on complications experienced by patients more comprehensively. Furthermore, as most of the studies were at the feasibility/exploratory state, it was not reported whether ECoG and impedance monitoring improved survival rates, aided a larger extent of resection and helped preserve neurological functionality. While at this stage these techniques may not replace the diagnostic yield of tools such as MRI, they could offer direct assistance during tumour resection. There was also no information on the operating surgeon experience and their likelihood of adopting the technique. Commenting on this data in future studies could support the incorporation of ECoG and impedance monitoring in the surgical workflow.

## Limitations

This systematic review was subject to limitations. As only English publications were included, this may have led to language bias with potentially relevant studies excluded. Additionally, conference proceedings were not included as this review focused on techniques (which were not adequately described in abstracts). The methods for reviewing grey literature may also have been limited. To keep the review focused, results pertaining to non-tumour pathologies such as arteriovenous malformations were excluded, however this also meant that data that could not be discerned between tumour and non-tumour pathologies was excluded, introducing potential selective reporting bias. Moreover, it is important to note the presence of time-period bias, which we could not assess, as there have been changes in the histopathological diagnosis of tumours based on the WHO classification, throughout the years. Furthermore, as this review included studies with predominantly adult patients (≥ 18 years), results may not be applicable to the paediatric population, which presents additional considerations for neurophysiological intraoperative monitoring, whether in the awake or asleep setting. Lastly, this review is subject to publication bias as none of the included studies reported on negative findings.

## Conclusion

Positive results from previous studies suggest that multimodal recordings (ECoG and impedance monitoring) of brain tumour activity have the potential to contribute to collecting datasets with the potential to accurately identify tumour margins. We also recommend for more extensive standardised research, across different tumour subtypes, WHO grades, and locations, to delineate inter-tumoural differences and to establish best practices in the field. Due to the nature of these interventions, it is not possible to have a control group, however, future studies could account for this by enrolling larger patient groups.

## Supplementary Information

Below is the link to the electronic supplementary material.

Supplementary file1 (DOCX 38 KB)

## Data Availability

No datasets were generated or analysed during the current study.
